# Defining barriers and enablers for clinical pathway implementation in complex clinical settings

**DOI:** 10.1186/s13012-018-0832-8

**Published:** 2018-11-12

**Authors:** Mona Jabbour, Amanda S. Newton, David Johnson, Janet A. Curran

**Affiliations:** 1 Department of Pediatrics, Division of Emergency Medicine, Children’s Hospital of Eastern Ontario, 401 Smyth Road, Room W1415, Ottawa, ON K1H 8L1 Canada; 2 University of Ottawa, Ottawa, ON Canada; 3 Children’s Hospital of Eastern Ontario Research Institute, Ottawa, ON Canada; 4 Department of Pediatrics, Division of General Pediatrics, Faculty of Medicine and Dentistry, University of Alberta, Edmonton, AB Canada; 5 Departments of Pediatrics and Emergency Medicine, University of Calgary, Calgary, AB Canada; 6 Department of Physiology and Pharmacology, Cumming School of Medicine, University of Calgary, Calgary, AB Canada; 7 Alberta Health Services, Calgary, AB Canada; 8 School of Nursing, Faculty of Health Professions, Dalhousie University, Halifax, NS Canada; 9 Department of Emergency Medicine, IWK Health Centre, Halifax, NS Canada

**Keywords:** Implementation, Clinical pathways, Barriers and enablers, Theoretical domains framework, Emergency medicine

## Abstract

**Background:**

While clinical pathways have the potential to improve patient outcomes and reduce healthcare costs, their true impact has been limited by variable implementation strategies and suboptimal research designs. This paper explores a comprehensive set of factors perceived by emergency department staff and administrative leads to influence clinical pathway implementation within the complex and dynamic environments of community emergency department settings.

**Methods:**

This descriptive, qualitative study involved emergency health professionals and administrators of 15 community hospitals across Ontario, Canada. As part of our larger cluster randomized controlled trial, each site was in the preparation phase to implement one of two clinical pathways: pediatric asthma or pediatric vomiting and diarrhea. Data were collected from three sources: (i) a mediated group discussion with site champions during the project launch meeting; (ii) a semi-structured site visit of each emergency department; and (iii) key informant interviews with an administrative lead from each hospital. The Theoretical Domains Framework (TDF) was used to guide the interviews and thematically analyze the data. Domains within each major theme were then mapped onto the COM-B model—capability, opportunity, and motivation—of the Behaviour Change Wheel.

**Results:**

Seven discrete themes and 58 subthemes were identified that comprised a set of barriers and enablers relevant to the planned clinical pathway implementation. Within two themes, three distinct levels of impact emerged, namely (i) the individual health professional, (ii) the emergency department team, and (iii) the broader hospital context. The TDF domains occurring most frequently were Memory, Attention and Decision Processes, Environmental Context and Resources, Behavioural Regulation, and Reinforcement. Mapping these barriers and enablers onto the COM-B model provided an organized perspective on how these issues may be interacting. Several factors were viewed as both negative and positive across different perspectives. Two of the seven themes were limited to one component, while four involved all three components of the COM-B model.

**Conclusions:**

Using a theory-based approach ensured systematic and comprehensive identification of relevant barriers and enablers to clinical pathway implementation in ED settings. The COM-B system of the Behaviour Change Wheel provided a useful perspective on how these factors might interact to effect change.

**Trial registration:**

ClinicalTrials.gov, NCT01815710.

## Background

The evidence-to-practice gap remains a healthcare challenge [1–8]. While knowledge syntheses and clinical practice guidelines (CPGs) have emerged as rigorous means to translate and make research more accessible for practitioners, these are not sufficient to change practice behaviour, especially in complex settings such as the chaotic environment of an emergency department (ED) [9, 10]. As a tool to operationalize best evidence recommendations and CPGs into an accessible bedside format for healthcare teams, the clinical pathway (CP) is a potentially important strategy for effective knowledge translation. CPs have the capacity to promote standardized evidence-based practices, patient safety, and health system efficiency [11–19]. CPs can reduce a clinician’s cognitive load (mental effort) to allow them to focus on more complex, thought-requiring activities [20]. A well-designed CP can support healthcare teams to deliver key management priorities in a timely manner. As a result, CPs are increasingly used in healthcare settings and recommended by broader healthcare systems internationally as a form of quality improvement [21, 22]. However, CPs are infrequently used outside of academic or large EDs, and their impact in community settings remains unknown [21]. In the field of emergency medicine, there is exceeding demand to achieve improved wait times and patient throughput [23]. This pressure threatens the safe and quality care that is important to ED healthcare providers who must contend with a diverse population of varying ages, medical conditions, and treatments.

Most CPs are developed internally within a hospital. As such, CP quality may be limited by lack of rigour and expertise locally in interpreting best evidence for incorporation into that pathway. Expert-developed CPs, created by multidisciplinary teams of clinicians, researchers and end-users at broader regional and national levels offer an opportunity for high-quality content and professional design. Expert CPs can be a means to ensure the standard of care is provided across different jurisdictions. However, while contextual knowledge may facilitate local uptake of internally developed CPs [11, 24, 25], expert-developed CPs may pose greater challenges with local implementation.

Although CPs have the potential to link evidence to practice via integration of CPGs into local systems and improve patient outcomes while decreasing health costs, their true impact has been limited by variable implementation strategies and sub-optimal research designs [26–30]. Evidence is limited on the optimal process of CP implementation [27, 29, 31]. Expert CPs cannot simply be imposed, and implementation at the local level, especially in community ED settings, can be challenging [32, 33]. An effective implementation strategy requires a thoughtful understanding of current and anticipated obstacles [34]. Because ED-based CPs generally involve the full health team, hospital contextual issues and ED team dynamics may be key factors. Evidence-based strategies used to implement CPGs may not be sufficient to promote CP adoption in ED settings, as the complexities of behaviour change among health providers are compounded by organizational and system-level barriers. Effective strategies for implementing CPs in these settings are largely unknown [27], and this knowledge gap must be addressed before their full impact can be realized.

Some have advocated for the use of models and change management theories to design successful CP implementation [35]**.** In a retrospective study of managerial factors associated with failed and successful CP implementations, key success factors included alignment of goals, choice of CP, and leaders’ roles [36]. However, few studies have prospectively tested and associated specific intervention components with successful implementations. Within emergency medicine [37], calls have been made for an increased focus on implementation science research to identify success factors for the implementation of evidence-based interventions. A recent systematic scoping review of implementation research in emergency medicine found that analysis of factors and use of theory to understand these factors occurred infrequently in the papers reviewed [38]. Rather than the current best-guess approach to implementation, the authors recommend systematic assessments of critical barriers and enablers, guided by theory-based approaches to understand key behavioural determinants that will inform targeted intervention design. We found one such paper, although in a different field, that identified five themes of potential implementation barriers and enablers for a CP to screen for anxiety and depression in cancer care [39]. Clearly, more of this work is needed in emergency medicine and other fields. This study uses implementation theories to understand clinical practice change in complex and dynamic environments, such as the ED setting.

We are currently evaluating the implementation of two expert-derived emergency care CPs: (i) asthma and (ii) vomiting and diarrhea (V&D) in children in two separate cluster randomized controlled trials (RCTs) being conducted in 15 community EDs across Ontario, Canada [40]. We began with a set of core components, based on our clinical and leadership experience in outreach initiatives [41], and sought to tailor our intervention strategy to address relevant issues among the study sites. The aim of this research was to identify a comprehensive set of factors perceived to influence the implementation of asthma and V&D CPs within community ED settings. This pre-implementation study documents foundational research prior to implementation of the CPs in the 15 EDs.

## Methods

### Design and participants

We conducted a descriptive, qualitative inquiry to understand barriers and enablers to implementing the CPs. The study sites included 15 community EDs across Ontario, Canada, representing different annual patient census volumes and urban/rural contexts. Each site identified one ED nurse and one ED physician (‘champion pairs’) to liaise with our study team, recruit participants, and assist with local implementation of the CP. Potential participants included administrative leads (i.e., ED director, manager, or hospital lead) and ED physicians and nurses. The study was approved by the Research Ethics Boards of the Children’s Hospital of Eastern Ontario and each of the 15 partner hospital organizations.

### Data collection

To develop a rich data set about potential barriers and enablers within these complex systems, we took a broad approach by collecting data using three different, but complementary, strategies. For each strategy, we created distinct data collection guides informed by the Theoretical Domains Framework (TDF) [42], which represents a comprehensive set of 14 theoretical domains derived from behaviour change theories and constructs. The TDF provides a useful approach to explore individual and system-level factors that affect behaviour change strategies [43].

#### Strategy 1

A daylong project launch meeting, involving champion pairs from all 15 ED sites, was held in September 2013 in Toronto, Ontario. During this meeting, we held a 90-min mediated group discussion including all site champions. A semi-structured question guide was used to solicit information regarding potential barriers and facilitators to implementing one of the CPs in their respective EDs. The items were based on our previous CP implementation experience [41] and included issues such as concerns about and foreseeable barriers to implementing such an initiative in their EDs. A trained qualitative research coordinator (KR) facilitated the discussion and several team members took detailed notes. The discussion was audio-recorded and later transcribed, along with the field notes.

#### Strategy 2

Site visits were conducted at all 15 EDs between August 2013 and January 2014 and included a discussion with the designated site champions and a diverse sample of ED physicians and nurses. A semi-structured guide was created to organize and structure data collection at each site. This guide included general demographic, staffing, and organizational issues, such as experience with standardized protocols, reaction to pediatric patients, and anticipated CP implementation barriers. To ascertain typical assessment and treatment practices for pediatric patients with asthma or V&D in their ED, a think aloud question guide was also created to “walk through” a scenario with each nurse and physician interviewed. For example, for a child presenting with respiratory difficulty, the triage nurse is asked whether any standardized tools are used to assess severity. The ED treating nurse is asked about comfort with initiating treatment prior to physician assessment, and the ED physician is asked about confidence in the nursing team to initiate this therapy. The principal investigator, a pediatric emergency physician (MJ), and a pediatric emergency nurse study coordinator (DD) conducted the interviews at each site using the question guide and took detailed notes during the visit. Each visit also included a physical tour of the ED to assess design and workflows, with emphasis on pediatric patients.

#### Strategy 3

Key informant (KI) interviews were held with an ED or hospital administrative lead from each site. All interviews were guided by a 31-item open-ended questionnaire based on the TDF, with questions such as (i) how confident do you feel in your hospital’s ability to implement a clinical pathway to manage pediatric asthma?; and (ii) what do you think are the consequences for your hospital of using the clinical pathway to treat pediatric asthma in the emergency department? We piloted the interview guide with nearby community ED leads not involved in this study. All telephone interviews were conducted by telephone by the study coordinator (KR) from October 2013 to February 2014. Each participant was made aware of the study aim. In recognition of participants’ time and expertise, each was offered a $50 honorarium.

The audio files from Strategy 1 and 3 interviews were transcribed verbatim by an external contractor. The transcripts were then anonymized and imported into NVivo 10 [QSR International] for analysis, along with the transcribed field notes taken from the mediated group discussion and site visits.

### Data analysis

Analysis followed four steps detailed below. During the study, the principal investigator (MJ), study nurse coordinator, and qualitative research coordinator worked closely with each of the sites. Study co-investigators were later engaged in the data analysis; these individuals were not known to or familiar with the staff or environmental issues at these study sites.

#### Coding

The research coordinator (KR) reviewed the transcripts and notes to gain a sense of the data, then used a directed content analysis approach [44] to code the data using the 14 domains of the TDF as a coding framework. The research team met several times to refine categories and clarify any issues. The second coder (DD) independently coded 30% of each type of documents to ensure inter-rater agreement. Using an iterative process, the coders met four times to review coding, resolve any problems or discrepancies, and reach consensus on the coding. When necessary, one of two investigators (MJ, JC) was involved to resolve any disputes. The coding was then revised to reflect these discussions. Memos were used to record relevant discussions and coding notes.

#### Generating belief statements/themes

KR then examined data coded into each of the domains to generate common belief statements that suggested a problem and/or influence on CP implementation [45]. These summaries were reviewed for accuracy by the second coder (DD) and refined further by two of the investigators (MJ, JC). These were then grouped into broader themes/sub-themes of barriers and enablers for CP implementation in these ED sites.

##### Linking barrier/enabler themes to relevant theoretical domains

Consistent with previous studies [43, 46, 47], TDF domains were deemed important to CP implementation if (i) they contained multiple themes/subthemes, (ii) conflicting themes/subthemes existed for that domain, or (iii) the themes within the domain were identified as having a strong impact on CP implementation (crossed multiple domains). These criteria were reviewed concurrently in a series of web-conference meetings involving three of the investigators (MJ, JC, MN). The principal investigator (MJ) created definitions for each theme and subtheme that were reviewed and approved by two other co-investigators (JC and MN). MJ identified a set of illustrative quotes for each subtheme from the coded transcripts. Using a consensus process, where discussions were held to review and resolve differing perspectives, we then selected the most salient of these quotes for each theme, highlighting subthemes and relevant TDF domains within each theme. For additional rigour, the co-principal investigator (DWJ) joined the process to validate and further refine the findings. Using a consensus process through a series of webinar meetings, one to two exemplar quotes were selected for each subtheme. Webinar discussions were audio-recorded for later reference.

#### Mapping themes

As a final step, the identified domains and cross-cutting major themes were mapped onto the Behaviour Change Wheel (BCW), a synthesis of 19 behaviour change frameworks from a range of disciplines and approaches [48]. Within the BCW, three sources—*capability*, *opportunity*, and *motivation*—interact to generate *behaviour* (COM-B system) and specific TDF domains have been described to align with these sources of behaviour [49]. This structured behavioural system assists with linking relevant themes identified through TDF analysis in Step 3 with intervention functions and policy options that may be useful to consider in designing an implementation strategy.

## Results

### Sample characteristics

Demographics of the 15 partner ED sites are summarized in Table 1. These included a range of total annual and pediatric volumes, community types, and access to Pediatric consultants. Sources for each data collection strategy are shown in Table 2. Participants from all 15 study hospitals were represented at the mediated group discussion, including the ED physician and ED nurse champions from each site. Three study hospitals had more than one campus; two of these included different administrative leads. In total, 17 key informant interviews were conducted, with a duration between 15 and 60 min (mean = 28.4; min SD = 11.2 min). No repeat interviews were conducted. Site visits were conducted at each hospital and involved discussion with one or both site champions, as well as ED physicians and nurses currently on duty. Many consistencies were found across the three data collection strategies, such as perceived value of these standardized tools, relative lack of comfort and expertise with pediatric patients, and challenges of the chaotic ED environment and many competing priorities. However, some differences were also found across these strategies. Comments expressed at the mediated group discussion were more supportive and consensus-building in nature, while the KI interviews expressed more challenges as seen by ED leaders. Data from the site visits provided variable front line perspectives from ED staff and physicians, tending to reflect cultural issues within that ED.

**Table 1:** Site demographics

Site Number	Community type	Annual census^a^	Pediatric census^a^	Access to pediatric consultant
Very high volume emergency departments				
3	Urban	76,349	14,264	In-house
4	Urban	65,762	19,083	In-house
10	Urban	67,810	13,584	In-house
13	Urban	107,436	13,087	In-house
15 + 16 (2 campuses)	Urban	122,251	39,971	In-house
17	Urban	58,884	18,959	In-house
High volume emergency departments				
2	Rural	34,301	6885	In-house
5 + 12 (2 campuses)	Rural	48,874	8319	By phone
6	Urban	33,011	12,210	In-house
7	Rural	45,136	9736	By phone
9 + 18 (2 campuses)	Rural	44,836	7538	By phone
11	Rural	45,644	8904	By phone
14	Rural	32,661	7029	By phone
Medium volume emergency departments				
1	Rural	22,744	8853	By phone
8	Rural	25,805	4568	In-house

^a^Census data based on the following dates: April 1st, 2011 to March 31st, 2012

**Table 2:** Sources of data

Strategy		Participants		
		RNs	MDs	Hospital administrator
1	Mediated group discussion (project launch)	18 (RN site champions	15 (MD site champions)	2
2	KI interviews *N* = 17 interviews	6 RN managers/directors	7 ED chiefs/medical directors 1 MD champion	3
3	Site visits *N* = 15 visits	30^a^ (1 triage RN, 1 treating RN per site)	15^a^ (1 MD per site)	4

*RN* registered nurse, *MD* medical doctor, *KI* key informant

^a^RN and MD site champions also participated

### Relevant TDF domains: barriers and enablers to implementing the asthma or V&D CPs in EDs

Seven major themes and fifty-eight sub-themes of barriers and enablers were identified as relevant to CP implementation, as shown in Table 3. These include the following themes: (1) CP Tools and Standardization; (2) Pediatric/Patient-specific Issues; (3) Professional Issues; (4) Team Dynamics; (5) Strategies for Success and Sustainability; (6) Hospital Resources and Processes; and (7) Quality and Process Improvement. Within the first two themes, three distinct levels of impact emerged: (i) the ED health professional at an individual level, (ii) the ED Team, and (iii) the broader hospital context. Sub-themes clustered most within the first theme (CP Tools and Standardization), occurring equally amongst each level of impact. The themes Team Dynamics and Hospital Resources and Processes had the next most frequent number of subthemes. The fewest number occurred within the Quality and Process Improvement theme.

**Table 3:** Major themes and sub-themes

Themes (sub-themes) and definitions		TDF domain
1. CP and Standardization		
Health Professional Level	CP quality: *confidence that CP is based on best available current evidence*	Beliefs about consequences
		Knowledge
	Awareness of and benefits of using this CP	
	Ability to follow CP and medical directives	Skills
	Sustained CP use: *sustained CP use questioned post-study*	Memory, Attention, and Decision Processes
	Perceived value of standardization: perception that standardization is good; improves health care. CP aids decision-making and will minimize errors	Social/Professional Role and Identity
		Memory, Attention, and Decision Processes
	New scoring tools: unknown scoring tools anticipated as difficult to remember components	Knowledge
		Memory, Attention, and Decision Processes
ED Team Level	*Experience with other CPs/standardized tools that can help with this implementation*	Knowledge
	Perceived value of evidence-based standardized practice: reception to standard work. Standardization is good; improves health care	Social/Professional Role and Identity
	ED impact: *postive and negative*	Beliefs about consequences
	General commitment to best practice and best patient outcomes: general commitment across ED team/hospital to quality and process improvement initiatives	Goals
		Intentions
	External social influences: impact of non-ED members (e.g., pediatricians) on CP use	Goals
		Reinforcement
	Experience for future improvement processes	Social Influences
Organizational Context Level	Ready access to CP Tools: accessibilty to CP tools	Behavioural Regulation
	User-friendly tools: *clear, easy documentation with minimal duplication*	
	Organizational reinforcement: CP might be helpful for sites with limited resources	
		Memory, Attention, and Decision Processes
		Reinforcement
	Hospital Impact: *postive and negative*	Beliefs about consequences
	Administrators’ commitment to CP implementation	Intentions
2. Pediatric/Patient-Specific Issues		
Health Professional Level	Knowledge and (lack of) experience in pediatrics may affect comfort with using the CP; may also create interest in the CP	Knowledge
		Skills
		Beliefs about capabilities
	Fear/anxiety with pediatric patients: generalized anxiety that pediatric patients deteriorate quickly. Peds patients generally have staff “at attention”	Emotion
ED Team Level	Benefits to patients: *positive patient benefits are motivating to staff and administrators*	Reinforcement
	Parental emotions: *parental emotions may heighten stress among ED team*	Emotion
	Impact on patient care: *using the CP will positively impact patient care.*	Beliefs about consequences
Organizational Context Level	Benefits to patients	Beliefs about consequences
	Pediatrics factors	Environmental Context and Resources
3. Professional Issues		
Intrinsic rewards: *potential impact on job satisfaction, professional well-being*		Reinforcement
Scope of RN vs MD practice: *CP shifts roles and scope of work: RNs can do more, less for the MDs to do*		Social/Professional Role and Identity
Workload capacity: *impact of other work on ability to implement/use the CP*		Beliefs about capabilities
Threats to autonomy or decision-making: *perceived threats to autonomy/ decision-making among MDs with use of the CP; opporutnity for input on CP; MD skepticism. Potentially offensive to clinicians to assume decision-making assistance is needed*		Social/Professional Role and Identity
		Memory, Attention, and Decision Processes
		Behavioural Regulation
Staff/physician ED experience: *inexperienced RNs, part-time and locum MDs may impact ability to follow directives, CPs; may facilitate implementation since minimal practice change is required*		Skills
		Beliefs about capabilities
		Environmental Context and Resources
Unfamiliarity with the CP: *generalized concern about doing things differently, learning about a new CP*		Emotion
		Memory, Attention, and Decision Processes
Cognitive demands: *until CP is engrained in practice, more cognitive demand and attention required.*		Memory, Attention, and Decision Processes
Competing priorities: *many competing priorities threaten attention to CP use; CP topics not priority for EDs*		
4. Team Dynamics		
Confidence in Interdisciplinary Capabilities: *Perceived MD confidence in RN’s abilities; RN confidence in MD’s abilities*		Beliefs about capabilities
Confidence in team: *confidence in hospital/ ED team ability to implement/use the CP, including impact of positive past experiences*		Optimism
		Beliefs about capabilities
		Goals
Change fatigue: frustration/burnout with change among ED teams/hospitals may impact this CP implementation		Emotion
		Memory, Attention, and Decision Processes
Competing ED priorities: many competing ED priorities threaten attention to CP use; CP topics not priority issues for EDs		Memory, Attention, and Decision Processes
		Environmental Context and Resources
Concern that CP use may decrease during busy shifts or challenging periods, which are when the CP can be most helpful.		Environmental Context and Resources
		Memory, Attention, and Decision Processes
Formal/informal champion: local champion actions influence use of CP, directly and indirectly		Reinforcement
Adaptability, resistance, and buy-in: adaptability or lack thereof among staff to accept and adopt the CP		Social Influences
Interdisciplinary influences: impact of RNs on MD practice behaviour, and vice-versa		Social Influences
Conformity/conflict: pressures within the ED team to conform; conflicts within team		
Staff size: impacts ability to introduce and adopt the CP		Environmental Context and Resources
		Optimism
5. Strategies for Success and Sustainability		
*Strategies used to impart relevant knowledge, skills; reinforce and regulate behaviours for CP use*		
Education strategies: -In-shift training -Web modules -Professional education credits; huddles; narratives (stories); interdisciplinary training sessions; case examples; side-by-side modelling)		Knowledge
		Skills
		Reinforcement
		Behavioural Regulation
Communication: *use of communication to share knowledge, reinforce, and regulate behaviour*		
Audit and feedback: *use of audit and feedback to share knowledge, reinforce, and regulate behaviour*		Behavioural Regulation
		Reinforcement
Triggers/reminders: *use of triggers and reminders to reinforce appropriate CP use* -Posters; pocket cards; triage triggers; site champion/ super-user(s) -Integrate into existing technologies		Reinforcement
		Memory, Attention, and Decision
		Behavioural regulation
Input: opportunity to provide input on CP tools is likely to affect its use among staff (esp. MDs)		Behavioural regulation
Recognition: *recognition to highlight those appropriately using the CP*		Behavioural regulation
6. Hospital Resources and Processes		
Staffing: *presence of stable and committed staffing group with appropriate supports*		Environmental Context and Resources
IT support: *support for IT related aspects of CP access and functioning*		
Organizational priorities: *priority initiatives at organizational level*		
Physical design, space: *physical setup and use of space in the ED*		
Drugs, equipment: *access and availability of drugs, equipment related to the CP*		
Approval committees: *processes and delays for CP approval from various hospital committees*		
Multi-site hospital campuses: *several hospitals have multisite campuses with*		
Setting: *impact of urban* vs *rural setting*		
Funding: *pressures related to ED Wait Times funding incentives*		
7. Quality and Process Improvement		
General commitment to best practice and best patient outcomes		Intentions
		Goals
Impact of positive past experiences		Optimism

*CP* clinical pathway, *ED* emergency department, *RN* registered nurse, *MD* medical doctor

Each subtheme was assigned to one or more relevant TDF domains, resulting in a total of 86 domain assignments across the subthemes. All 14 domains were represented, but to varying extents. The most frequently occurring TDF domains were *Memory*, *Attention and Decision Processes*, which was relevant within four different themes, and *Environmental Context and Resources*, which clustered heavily within the Hospital Resources and Processes theme. The *Behavioural Regulation* and *Reinforcement* domains were also frequently identified.

### Mapping themes onto the behaviour change wheel

**Fig. 1:**
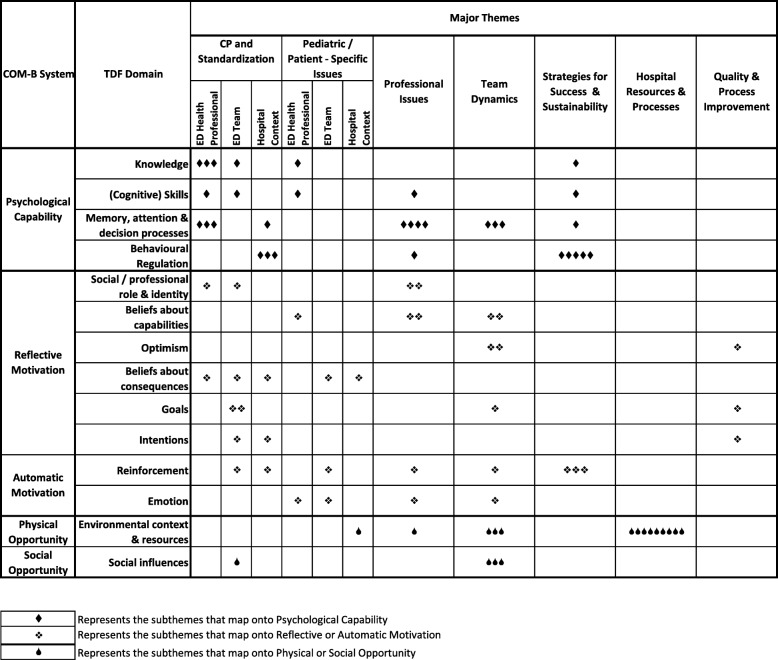
Summary of major themes by TDF Domains and corresponding COM-B intervention factors. TDF Theoretical Domains Framework, COM-B Capabilities, Opportunities, Motivation-Behaviour

Figure 1 illustrates how the full set of 86 TDF domains relating to the barrier and enabler subthemes maps onto the corresponding COM-B intervention factors. Some themes, such as the Quality and Process Improvement and Hospital Resources and Processes, mapped exclusively to specific intervention factors, namely Reflective Motivation and Physical Opportunity, respectively. Other themes were distributed more evenly across the factors. Our findings are further described below based on the Behaviour Change Wheel COM-B model.

### Capability

#### Knowledge, skills, memory, attention and decision processes, behavioural regulation

Participants identified the importance of knowing the CP exists and believing that it is high quality and based on best available evidence*:* Equally important, the CP tools needed to be easily accessed within the ED and user-friendly with minimal duplication efforts.

*“And so I think simplicity is very important.”* [KI Interview Site 3].

*“The docs tell me they don’t want to be writing it twice because you write the orders right on the chart right when you see the patient, and then you got to go and get some order set and start ticking off other things again.”* [KI Interview Site 3].

Various ongoing and competing priorities within the ED threatened use of the CP; until it is ingrained in practice, there will be more cognitive demands with using versus not using it. Moreover, because in some settings, there were relatively infrequent opportunities to use the CP, staff needed to remember to use it. However, several educational and behavioural regulation strategies were identified that could be helpful.

*“Right and if there’s data or graphs to show a decrease in length of stay, an increase in health of the child, the effects of using that pathway, that’s always good to show docs because they’re data driven but we’re … our department is very data driven so I mean those kinds of education pieces are valuable”* [KI interview site 10].

Experience with other pathways and standardization tools were thought to be helpful for implementation: *“I feel fairly confident; we’re a group that’s quite familiar with change. We’ve done several initiatives in the past that looked at process improvement, so I don’t foresee any issues with the adoption of it.”* [KI Interview Site 17]. Additionally, several conflictual issues were identified related to Capability. First, while some believe that utilization of the pathway among physicians can be facilitated by simply handing this to them, others expressed more challenges with consistency given the infrequent and variable presence of some physicians.

*“I think some of them will be brought on board, again, just by the fact that the nurse will have handed them the protocol and so it’s right there in front of them so they’re going to use it because it’s there.”* [Mediated Group Discussion].

*“Our docs typically may only spend one, maybe two shifts a week in the emerg department. And so we may have a locum who spends only one shift a month in our hospital. So they are a much harder group to get consistent because they’re just not there enough.”* [KI Interview Site 7].

Secondly, due to ubiquitous staffing pressures, the reality of inexperienced RNs and part-time or locum MDs was felt by some to limit their ability to use the CP. However, their lack of ingrained practices might facilitate CP uptake if this was understood as the standard within a given ED. Finally, the relative infrequency of pediatric visits resulted in less opportunity for use and comfort with the CP. In contrast, a CP tool was deemed by some as even more helpful given this relative lack of pediatric experience.

### Motivation

#### Social/professional role and identity, beliefs about capabilities, beliefs about consequences, optimism, goals, intentions, reinforcement, emotion

Participants expressed a strong commitment to best practice and best patient outcomes. *“If that is best practice for the patient we’re all about giving best practice and high standards of care to the patients so that’s the main point right there.”* [KI Interview Site 10]. Specific commitment was noted at an administrative/leadership level to do what is required for successful CP implementation*: “… because it comes in surges and it can actually slow a department down drastically if you have an onslaught of patients with the same presentation and you have a team of doctors who each decide to treat it differently.”* [KI Interview Site 13].

Participants also frequently described a fear of pediatric patients, as they can deteriorate quickly, and as a result, have the staff “at attention”. This was compounded by the associated parental emotions and expectations. Hence, staff and physicians were interested in a CP tool that guided the care of pediatric patients.

*“[There is a] higher level of alertness for pediatric patients. They compensate until they get very sick; [they can] deteriorate quickly.”* [Site 11, site visit].

*“My personal view is that because of the complexity and the variability of a pediatric patient population, a pathway is very helpful. Because it can … it’s easy to get off course when you have huge variability and it, actually the pathway can kind of hold you to best practice and hold you to next steps.”* [KI Interview Site 4].

*“I think people are pleased that kids feel better and so … that sort of loops back on their decision making to continue to do it… I mean it’s re-evaluated on a patient to patient basis and I think people feel generally positive about the experience and so we keep using it.”* [KI Interview Site 14].

Conflictual issues were also identified. The CP shifts the scope of work within the ED team, with more responsibility for RNs. This was perceived favourably among RNs and many MDs. However, the CP was also seen to threaten autonomy and decision-making among MDs, especially when it is viewed cynically as “cookbook medicine”: *“So, there may be some physicians who cannot relinquish that sense of responsibility to the triage nurse staff that they are capable of doing … assessing the child properly. But I have great faith in my nursing staff. You might get those two different opinions.”* [KI Interview Site 6].

While positive patient benefits were highly motivating to ED staff and physicians, they were offset by the competing priorities and change fatigue present in many ED settings. It was also felt that the CP can result in positive impacts such as wait time metrics, patient satisfaction, and outcomes. However, concern was raised that some metrics may not improve or become worse. Finally, participants perceived conflicting confidence in interdisciplinary capabilities to use the CP, including physician concern with nurses’ ability to use the medical directives and nurses concern that physicians would not follow the CP.

### Opportunity

#### Environmental Context and Resources, Social Influences

Issues related to hospital resources and processes were frequently cited as relevant to potential success with CP implementation. These included committee approval processes, which can introduce lengthy delays, staffing issues, competing organizational priorities, physical design and space, and funding incentives such as the ED wait-times initiative that involves all the study hospitals. Again, competing ED priorities were viewed as a potential threat to CP use, especially in hospitals where the CP condition was not deemed a priority issue.

*“So, I believe when the organization started looking at standardizing practices, they looked at our top twenty CMGs [case mix groups], case management groupings and this was … pediatrics was not one of them.”* [KI Interview Site 9].

Challenging periods, such as high patient volumes or acuity, were also seen as threatening for CP use although several participants acknowledged that it is during these times that CPs can be most helpful. Some leaders viewed this planned implementation as a helpful experience for successive implementation initiatives. Finally, interdisciplinary influences and pressures for conformity within a team were also noted factors in CP adoption within that team. Participants expressed how nurses impact physician behaviour and vice versa, especially related to their adaptability, buy-in, or resistance in accepting and adopting the CP.

## Discussion

Using a multi-pronged qualitative approach in 15 hospital settings, we identified a comprehensive set of barriers and enablers that could affect CP implementation in community EDs. These factors are clustered within seven distinct themes, including (1) CP Tools and Standardization; (2) Pediatric/Patient-Specific issues; (3) Professional Issues; (4) Team Dynamics; (5) Strategies for Success and Sustainability; (6) Hospital Resources and Processes; and (7) Quality and Process Improvement. Additionally, three distinct levels of impact were identified across two of the themes, namely the ED health professional, ED team, and the hospital context levels, while the remaining themes clustered more within a specific level.

Our findings suggest that successful CP implementation in these complex clinical settings requires addressing barriers and enablers at multiple levels, individual providers (frontline and leadership), ED health teams, and the broader hospital context. Moreover, a systematic approach attending to all three levels of impact will be important. A motivated and well-intentioned professional is not sufficient if the team is not interested, or if the system does not support the implementation. Similarly, the system cannot easily push a CP if the individual professionals or team culture do not buy into it. In comparison, the implementation literature regarding clinical practice guidelines (CPGs) has identified dissemination, education and training, social interactions, and decision-support systems as successful strategies [50]. Similar to our findings, CPG implementation is more likely to be effective with the use of a multifaceted and strategic approach that addresses the context and identified barriers [51].

The factors identified in our study can inform CP intervention efforts by others by providing specific issues for consideration at each of these levels. We found that beliefs about the consequences (for patient/family) may also be strong motivators for implementing pediatric CPs. Patient-mediated interventions [52], such as the availability of brochures about specific CPs in the waiting room, might be used to educate and engage families to seek out CP-based care. The relative discomfort with managing potentially sick pediatric patients was ubiquitous. Participants frequently described their desire to provide quality care to children and trusted the CP to guide their care and ensure it is based on best evidence. Motivation may be diminished to implement CPs involving adult-related conditions, where providers feel they have more expertise.

The Theoretical Domains Framework (TDF) was useful to inform our data collection and analysis, providing a comprehensive approach to ensure relevant issues are raised. The TDF also provided a helpful structure in initially coding our data. However, the coding system required further modification to reflect the distinct themes deemed relevant to future CP implementation plans. This led to a clearer contextualization and more practical potential application of the relevant factors. The TDF is recognized as being more helpful for individual level change [43]. Given the complex and multilevel interactions involved in CP implementation, we found the COM-B system of behaviours influenced by capability, opportunity, and motivation [39,49,] to be more helpful in capturing relevant factors within a system. As per this model, a successful behaviour change intervention will need to change one or more of the following components: *Capability*, the ability to engage in thought or physical processes necessary for the behaviour; *Opportunity*, environmental or social factors that influence behaviour; or *Motivation*, the conscious belief and the unconsciously held emotions that direct behaviour. Our analysis identified elements across each of these components that will serve as important targets for our implementation strategy.

Several features strengthen our study. With representation from 15 hospitals with a range of sizes, communities, and other priorities, we could comprehensively explore factors that will be generalizable to other ED settings. We could also draw from a variety of sources within each hospital, including site champions, administrative leads, and front line ED nurses and physicians. Collectively, these provide a broad set of perspectives on the relevant factors affecting CP implementation within a given hospital. Finally, our study used theory to identify important barriers and enablers to CP implementation and to further conceptualize the findings.

This study is limited by some common factors well known to implementation research. Because site recruitment for CP implementation was voluntary, those agreeing to participate may have been the early adopters, have had positive experiences with process improvements, or were otherwise more motivated to take this on. As well, although we had representation from frontline clinicians, there may have been an oversampling among participants of leaders and educators. As such, our findings may be an optimistic perspective of the willing. We may not have heard sufficiently from those more constrained by challenges in the clinical setting or unwilling to undergo practice changes. Finally, the KI interviews asked administrators to comment on issues at the health professional and ED team levels, as well as the hospital context. However, the TDF framework is designed to assess change factors at the individual provider level. As such, the key informants’ views may not accurately represent those of frontline clinicians.

This paper is unique in its use of implementation theories to understand clinical practice change in a complex and dynamic environment, such as that of the hospital ED setting. There is currently a dearth of implementation evidence in these types of settings. The barriers and enablers identified in this study are important to the eventual success of our CP implementation strategy at these sites. These factors will also be helpful in articulating key steps and assessing fidelity with the actual implementation process. The findings from our study will help to guide further ED implementation research, adding to the body of implementation evidence in this unique setting.

## Conclusion

A comprehensive set of barriers and enablers to CP implementation within an ED setting has been identified from the perspectives of frontline ED health professionals and hospital leadership. Clustered within seven distinct themes and addressing three levels of impact—the ED health professional, the ED team, and the hospital context—we believe these barriers and enablers are essential considerations for a successful CP implementation strategy. The use of the Theoretical Domains Framework, to explore these factors, and the COM-B system of the Behaviour Change Wheel, to conceptualize how these themes might interact to effect change, has ensured a systematic approach in this process. These factors may form essential elements for a future CP implementation toolkit.

## Abbreviations

BCW: Behaviour change wheel
COM-B: Capability, opportunity, motivation-behaviour
CP: Clinical pathways
CPG: Clinical practice guidelines
ED: Emergency department
KI: Key informant (interviews)
RCTs: Randomized controlled trials
TDF: Theoretical domains framework
V&D: Vomiting and diarrhea
